# Multicystic tuberculosis pericarditis

**DOI:** 10.11604/pamj.2014.19.366.4666

**Published:** 2014-12-10

**Authors:** Hanane Hadj Kacem, Zakaria Bazid

**Affiliations:** 1 Radiology Department, CHU Mohamed VI, Medical School of Oujda, University Mohamed I, Oujda, Morocco; 2 Cardiology Department, CHU Mohamed VI, Medical School of Oujda, University Mohamed I, Oujda, Morocco

**Keywords:** Multicystic, rheumatic, tricuspid polyvalvulopathy

## Image in medicine

This case concerns a 43 year-old woman followed for rheumatic mitral-aortic and tricuspid polyvalvulopathy, for which she underwents a double mitral-aortic replacement with mechanical prosthesis and tricuspid annuloplasty in June 2009. She was also treated for cervical ganglion tuberculosis in 2011. The history of her illness had started three months earlier with the appearance of signs of right cardiac failure. Transthoracic echocardiography showed good functioning of the mitral and aortic prostheses, as well as of the tricuspid ring, and good ventricular systolic function, with a thick pericardium full of cysts, associated with signs of adiastole (A). A thoracic CT-scan showed abundant septated pericardial effusion, mullticystic, but without involvement of the pulmonary or ganglion parenchymas (B). An additional MRI was performed, revealing a view of pericardial constriction with numerous pericardial cysts, with intermediate signal intensity on T2-weighted black blood spin-echo images, high signal intensity on T2-weighted STIR sequences, with thickening of the pericardial layers (C), (D). A pericardial biopsy was carried out, which found a granulomatous inflammatory reaction with positive finding for BK virus. After 9 months of anti-bacillary treatment, the patient underwent partial pericardial decortication, in view of the multiple adhesions, associated with the establishment of a pleuro-pericardial window. Anatomopathological analysis of the pericardial fluid as well as of fragments of the pericardium collected, confirmed the diagnosis of a tuberculous pericarditis. The evolution was marked by a clear clinical improvement with disappearance of signs of cardiac failure, and with a weight gain of 16 Kg in 4 months. Postoperative ETT monitoring at 2 months, 4 months and 6 months showed complete disappearance of both the cystic pericardial effusion and the signs of a diastole.

**Figure 1:**
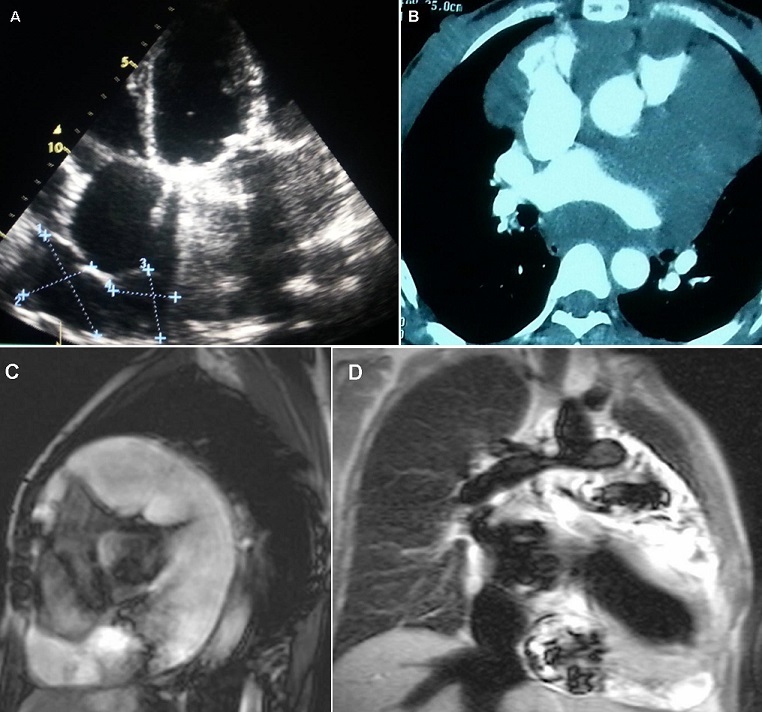
(A) axial Transthoracic echocardiography, Multiples cysts of pericardium, associated with signs of adiastole; (B) Axial contrast-enhanced CT scan of cheest. Abundant septated pericardial effusion, mullticystic extending into the mediastinum; (C) Heart MRI, T2-weighted black blood spin-echo images Pericardial constriction with numerous pericardial cysts, with intermediate signal intensity; (D) Heart MRI. T2-weighted STIR sequences Pericardial cysts, with high signal intensity

